# The neurotoxic effects of hydrogen peroxide and copper in Retzius nerve cells of the leech *Haemopis sanguisuga*

**DOI:** 10.1242/bio.014936

**Published:** 2016-03-02

**Authors:** Zorica D. Jovanovic, Marija B. Stanojevic, Vladimir B. Nedeljkov

**Affiliations:** 1 Department of Pathological Physiology, Faculty of Medical Sciences, University of Kragujevac, 34000 Kragujevac, Serbia; 2 Institute for Pathological Physiology, School of Medicine, University of Belgrade, 11000 Belgrade, Serbia

**Keywords:** Hydrogen peroxide, Copper, Leech, Antioxidants, Potassium current

## Abstract

Oxidative stress and the generation of reactive oxygen species (ROS) play an important role in cellular damage. Electrophysiological analyses have shown that membrane transport proteins are susceptible to ROS. In the present study, oxidative stress was induced in Retzius nerve cells of the leech *Haemopis sanguisuga* by bath application of 1 mM of hydrogen peroxide (H_2_O_2_) and 0.02 mM of copper (Cu) for 20 min. The H_2_O_2_/Cu(II) produced considerable changes in the electrical properties of the Retzius nerve cells. Intracellular recording of the resting membrane potential revealed that the neuronal membrane was depolarized in the presence of H_2_O_2_/Cu(II). We found that the amplitude of action potentials decreased, while the duration augmented in a progressive way along the drug exposure time. The combined application of H_2_O_2_ and Cu(II) caused an initial excitation followed by depression of the spontaneous electrical activity. Voltage-clamp recordings revealed a second effect of the oxidant, a powerful inhibition of the outward potassium channels responsible for the repolarization of action potentials. The neurotoxic effect of H_2_O_2_/Cu(II) on the spontaneous spike electrogenesis and outward K^+^ current of Retzius nerve cells was reduced in the presence of hydroxyl radical scavengers, dimethylthiourea and dimethyl sulfoxide, but not mannitol. This study provides evidence for the oxidative modification of outward potassium channels in Retzius nerve cells. The oxidative mechanism of the H_2_O_2_/Cu(II) system action on the electrical properties of Retzius neurons proposed in this study might have a wider significance, referring not only to leeches but also to mammalian neurons.

## Abstract (precis)

**Summary:** This characterization of the early changes in physiological membrane properties of Retzius neurons provides evidence for the oxidative modification of outward potassium channels.

## INTRODUCTION

Reactive oxygen species (ROS), such as superoxide anion (O_2_^•−^), hydrogen peroxide (H_2_O_2_), and hydroxyl radical (HO^•^), are produced continuously during normal cellular metabolism. There is growing evidence that H_2_O_2_ plays a role in normal cellular function and cell signaling (Dringen et al., 2005; Ray et al., 2012; Marinho et al., 2014), particularly in higher organisms. However, H_2_O_2_ shows a toxicological action, because it can produce the highly reactive HO^•^ and cause oxidative damage to biomolecules (Halliwell, 2006; Sabater and Martín, 2013). Compared with other ROS, H_2_O_2_ is relatively stable and also able of diffuse rapidly across cell membranes (Murrant and Reid, 2001). According to Halliwell et al. (2000), H_2_O_2_ may induce its deleterious action through direct oxidation of its substrate, or indirectly through its highly reactive byproduct HO^•^. Given its low reactivity, H_2_O_2_ does not readily mediate oxidative injury, unless exposed to transition metal ions that can catalyze transformation of H_2_O_2_ to the aggressive radical, HO^•^ (Cohen, 1994).

The transition metal ions and their complexes in lower oxidation states were found to have the oxidative property of the Fenton reagent, and, accordingly, the mixtures of these metals with H_2_O_2_ were named ‘Fenton-like’ reagents (Goldstein et al., 1993). Copper (Cu) is redox-active metal which is capable of inducing oxidative injury by two different mechanisms. Firstly, in the presence of H_2_O_2_, it can catalyze the formation of HO^•^ which can induce substantial damage of biomolecules by removing hydrogen or by addition to unsaturated bonds (Simon et al., 2004). Secondly, exposition of cells to increased level of copper diminishes intracellular glutathione content. Several authors have suggested that divalent copper [Cu(II)] reacts with H_2_O_2_ to produce HO^•^, which mediates oxidative damage. However, other researchers have disputed the formation of HO^•^ in reactions involving Cu(II) ions and H_2_O_2_, and the debate continues in the literature. Evidence of the role of copper in the production of ROS has been obtained mainly by *in vitro* study in which combinations of copper and a reducing agent were used. While a previous *in vitro* research has disclosed that Cu(II) ions are capable of reacting with H_2_O_2_ in a Fenton-like reaction (Gunther et al., 1995), it is still disputable whether this reaction occurs *in vivo*. The hydroxyl radical is small, highly mobile, water-soluble, and chemically the most reactive species of activated oxygen (Ayala et al., 2014). But, due to its very short half-life, it is effective only close to the locus of its generation. The short diffusion distance of the HO^•^ suggests that most metal-catalyzed oxidative modifications of proteins occur via the reaction of H_2_O_2_ with the sites of metals binding to the proteins (Sayre et al., 2005). HO^•^ is the most reactive and dangerous ROS since there are no enzymatic systems known to detoxify them. Due of its extraordinarily high reactivity, direct detection of HO^•^ in biological systems is very hard. For that reason, many researchers (Birinyi-Strachan et al., 2005; Huang et al., 2009a; Mokudai et al., 2012) used indirect methods for detecting and neutralizing this radical as well as antioxidants (mannitol, dimethylthiourea and dimethyl sulfoxide).

Electrophysiological analyses have shown that membrane transport proteins are susceptible to ROS. Considering neuronal network function, free radicals can attack ions channels either directly, or indirectly by causing peroxidation of membrane lipids (Carmeliet, 1999) and affecting associated signaling proteins (Hool, 2006). Oxidative modification of critical cysteine residues in redox-sensitive proteins has been proposed to constitute one of the major mechanisms that regulate protein structure and function (Zhang et al., 2006; Chung et al., 2013). In the light of this knowledge, surprisingly few experimental studies have focused on oxidative modification of K^+^ channels in nerve cells.

Having considered all the data noted above, the aim of this study was therefore to examine whether copper can enhance H_2_O_2_ toxicity and whether hydroxyl radical scavengers could protect leech Retzius nerve cells from toxicity induced by the H_2_O_2_/Cu(II) oxidizing system. The nervous system of invertebrates can be taken as a simple model for vertebrate brain studies in the aspect of synapse formation and plasticity (Schmold and Syed, 2012), and the neural basis of learning (Sahley, 1995). As multicellular organisms, the invertebrates represent a ‘simple model’, because their nervous systems are smaller and contain considerably fewer neurons than those found in brains of vertebrates. However, compared to vertebrate neurons, the structure and function of single nerve cells of invertebrates are equally complex (Burrell and Sahley, 2001; Bier and McGinnis, 2004). The main advantages of studying nerve cells in leech brain are the large sizes of Retzius nerve cells and their easy accessibility for electrophysiological recordings.

## RESULTS

Prior to experimentation, Retzius neurons were tested in order to check whether they would keep their electrophysiological properties intact for a minimum of 20 min, to ensure that changes on their intrinsic and firing properties were strictly induced by drug administration and not by the effect of time. At this control stage, the neurons tested (*n*=5) were recorded during 20 min or more and no significant differences in their membrane properties were observed. Once the control process was finished, we proceeded with the recording of the study sample. Monitoring of the electrical properties was carried out on the Retzius cells which show a stable membrane potential, firing rate and spike potential duration for at least 10 min. Most Retzius nerve cells had a resting membrane potential of −45 to −55 mV. Retzius neurons with a resting membrane potential less than −40 mV in normal leech saline were considered damaged and data from such cells were not analyzed.

### The effects of H_2_O_2_/Cu(II) on the resting membrane potential and spontaneous spike activity of Retzius nerve cells

**Fig. 1.**
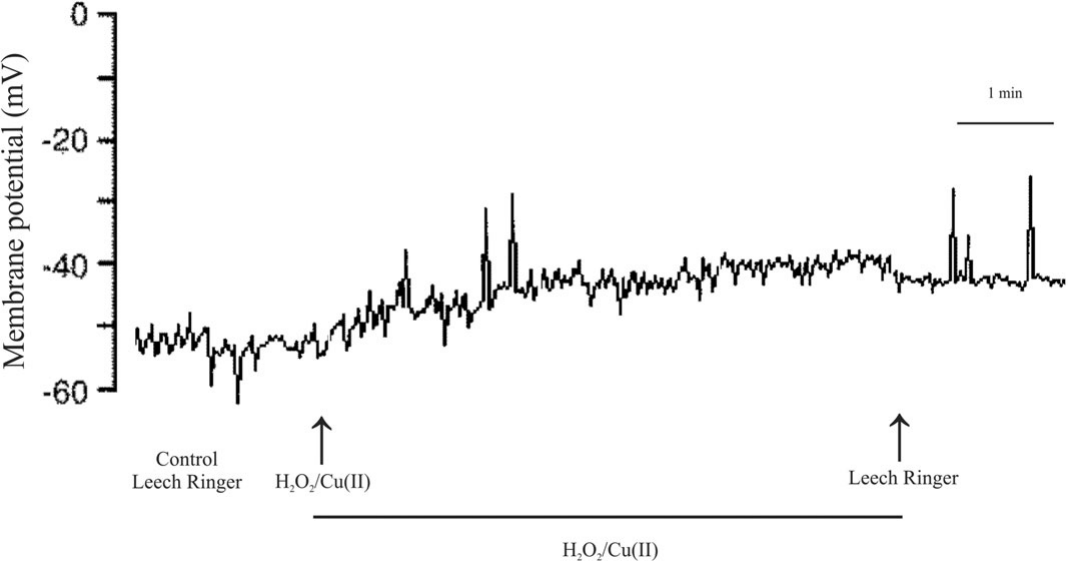
**The effect of hydrogen peroxide and copper on the membrane potential of Retzius nerve cell.** Extracellular application of 1 mM H_2_O_2_ and 0.02 mM Cu(II) depolarized the cell membrane potential of −53 mV by 9 mV.

In the first series of experiments, we investigated the actions of H_2_O_2_ in the presence of Cu(II) on the resting membrane potential of Retzius nerve cells. They showed a stable membrane potential of −46.75±2.28 mV (see Table 1), and spontaneous action potential of duration, amplitude and shape typical for Retzius cells (duration 7.52±1.25 ms, amplitude 45.94±3.86 mV) were generated at a low frequency (2.43±0.26 APs/s). Combined application of 1 mM of H_2_O_2_ and 0.02 mM of Cu(II) induced a slow and continuous membrane depolarization. As shown in Fig. 1 and Table 1, for all observations (*n*=20), membrane potential was significantly depolarized 5 min after drug administration. Values slightly increased after 15 and 20 min. The average change in membrane potential was 4.58±1.4 mV in the 5 min exposure, 6.84±1.9 mV in the 15 min, and 7.41±2.1 mV after 20 min administration (Table 1). For a sample neuron, Fig. 1 illustrates the effect of 1 mM of H_2_O_2_ and 0.02 mM of Cu(II) on the resting membrane potential of Retzius nerve cell. After a 20 min perfusion, we observed a membrane potential depolarization of approximately 9 mV.

**Table 1.**
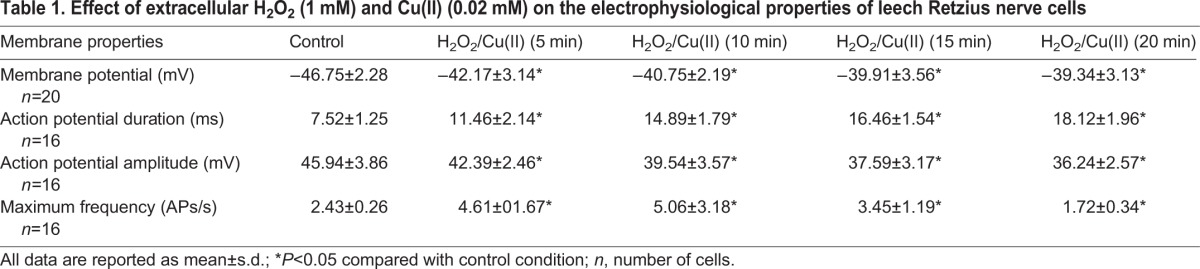
**Effect of extracellular H_2_O_2_ (1 mM) and Cu(II) (0.02 mM) on the electrophysiological properties of leech Retzius nerve cells**

Exposure of leech segmental ganglia to H_2_O_2_ (1 mM) and Cu(II) (0.02 mM) altered the duration and shape of the action potentials of Retzius nerve cells. We found that the amplitude of action potentials decreased, while the duration augmented in a progressive way along the drug exposure time (Table 1). With prior administration of H_2_O_2_/Cu(II), action potential duration (APD) amounted to 7.52±1.25 ms. On average, the prolongation of action potentials amounted to 10.6±1.17 (*n*=16, *P*≤0.01) after a 20-min exposure.

**Fig. 2.**
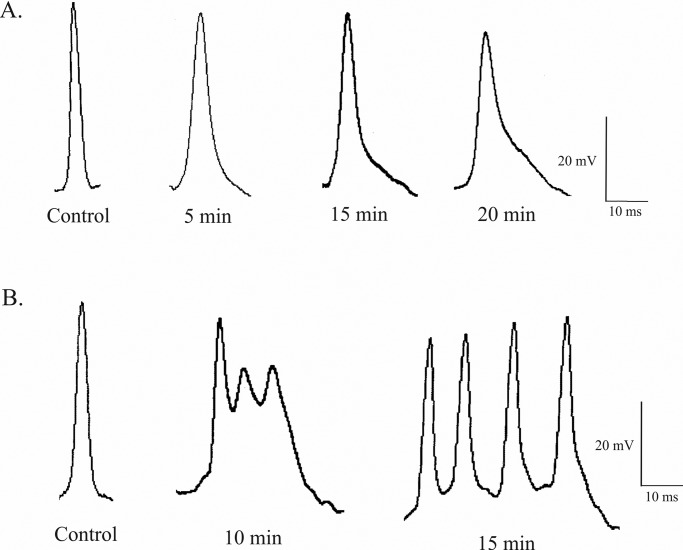
**The characteristic changes in the action potential of Retzius nerve cells produced by 1 mM H_2_O_2_ and 0.02 mM Cu(II).** (A) In the control leech Ringer, the Retzius cell have action potential of 48 mV with a duration of 7.4 ms. The amplitude of action potentials decreased, while duration increased in a progressive way along the drug exposure time. (B) Repetitive firing recorded in Retzius nerve cell 10 and 15 min after the application of H_2_O_2_/Cu(II).

In Fig. 2, we can observe the action potentials for a sample neuron that were obtained in control and experimental conditions. As seen in Fig. 2A, we found a widening effect in the spike. For a sample neuron, the duration of the action potential was 7.4 ms in control condition, 13.9 ms after 5 min, 17.5 ms after 15 min, and 19.4 ms after 20 min. Fig. 2A also demonstrates changes in the amplitude of the action potentials after H_2_O_2_/Cu(II) administration. The recordings show a progressive diminution of amplitude in the action potentials. In the control condition, amplitude measured 48 mV, it then decreased to 45 mV after 5 min and continued to diminish up to 42 mV after 15 min, and 37 mV after a 20-min exposure.

Our results demonstrate that most of the Retzius cells under study kept ability to discharge action potentials although their frequency decreased. Exposure of leech segmental ganglia to H_2_O_2_ and Cu(II) induced an initial excitation followed by depression of spontaneous electrical activity, and this effect was seen in 16 of 20 cells (Table 1). Furthermore, some of Retzius neurons lost their spontaneous activity. Thus, H_2_O_2_/Cu(II) led to the appearance of repetitive firing (Fig. 2B) only a few minutes after application of H_2_O_2_/Cu(II), which was followed by loss of excitability of the neurons. We found that 20% of Retzius cells under study showed a complete cancellation of discharge properties.

### The effects of H_2_O_2_/Cu(II) on the outward K^+^ current of leech Retzius nerve cells

**Fig. 3.**
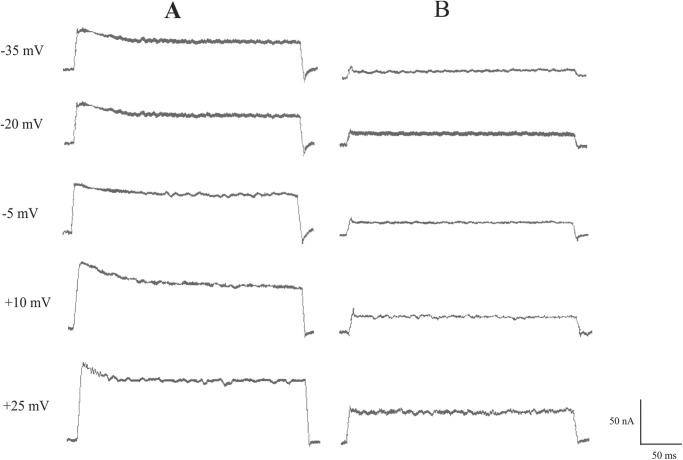
**The inhibition of the outward K^+^ current by H_2_O_2_/Cu(II).** Original tracings of the outward K^+^ current of Retzius nerve cell evoked by a 300 ms depolarizing pulse in the Tris Ringer (A) and presence of 1 mM H_2_O_2_ and 0.02 mM Cu(II) (B) for 10 min. At a clamp voltage of +25 mV, the fast and slow steady part of the K^+^ outward currents were reduced by 70% after exposure of the Retzius neurons to H_2_O_2_/Cu(II).

In order to examine the possibility that the broadening of action potentials of Retzius neurons were a consequence of the inhibition of the outward K^+^ current, needed for the repolarization phase of action potentials, we studied the effect of H_2_O_2_/Cu(II) on the outward K^+^ current. When Cu(II) (0.02 mM) was added to the leech Ringer, the concentration of the oxidant of 1 mM caused a strong inhibition of the K^+^ outward current. The typical response of a Retzius cell to H_2_O_2_ (1 mM) and 0.02 mM Cu(II) in an Na-free solution is depicted in Fig. 3. Outward K^+^ currents are generated by a series of depolarizing pulses, in the Tris Ringer (Fig. 3A) and after exposure of the Retzius neurons to 1 mM H_2_O_2_ and 0.02 mM Cu(II) for 10 min (Fig. 3B).

**Fig. 4.**
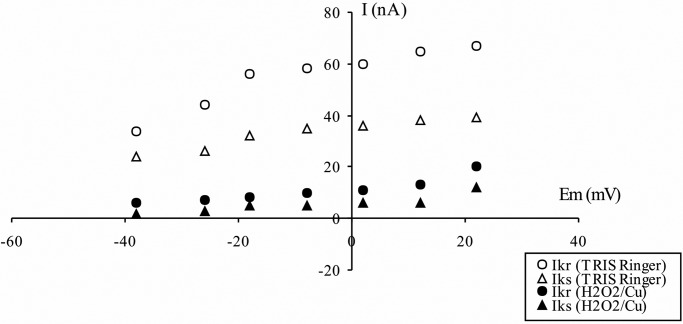
**The effects of H_2_O_2_/Cu(II) on the outward K^+^ current of Retzius nerve cells.** The current-voltage relationship at the peak of the K^+^ outward current (Ikr) and at the end of stimulation (Iks) in the absence (open symbols) and presence (solid symbols) of H_2_O_2_ (1 mM) and 0.02 mM Cu(II). Ikr, rapid outward K^+^ current; Iks, slow outward K^+^ current.

The current-voltage (I-V) relationships for the outward K^+^ current studied were generated by standardized pulse protocols and were obtained before and at various times after the addition of H_2_O_2_/Cu(II). Fig. 4 demonstrates the current-voltage relationship, separately, for the peak and the entrenched steady level of the K^+^ outward current. At the test potential of +22 mV, the fast and slow steady part of the K^+^ outward current dropped from 67 to 20 nA (70.15%) and from 39 to 12 nA (69.24%), respectively.

### The effect of the antioxidants on the prolonged action potentials and outward K^+^ currents of Retzius nerve cells

**Fig. 5.**
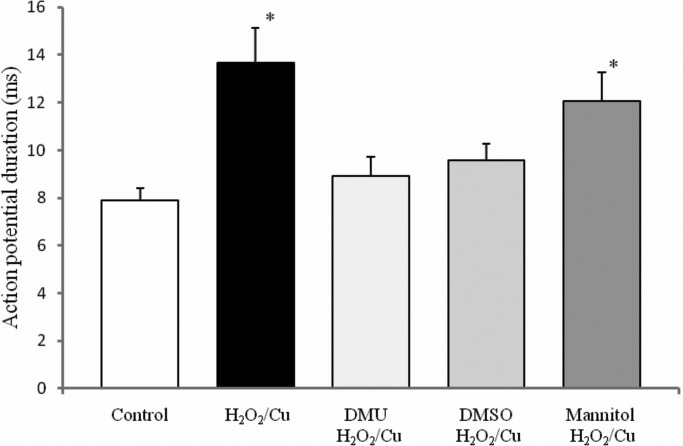
**The effects of the antioxidants on the duration of Retzius nerve cells' action potentials prolonged by H_2_O_2_/Cu(II).** The neurotoxic effect of H_2_O_2_/Cu(II) on spontaneous spike electrogenesis of the Retzius neurons was reduced in the presence of the dimethylthiourea (1 mM) and dimethyl sulfoxide (1%), but not the mannitol (5 mM). The measures are expressed as mean±s.d.; *P<0.05 compared with control condition.

To elucidate the mechanism underlying the effect of H_2_O_2_/Cu(II) oxidative activity, the actions of various antioxidants on H_2_O_2_/Cu(II)-induced changes were examined. The following experiments were carried out to investigate the possible contribution of HO^•^ production by the Fenton reaction to H_2_O_2_-induced electrical changes in Retzius neurons. The data presented in Fig. 5 shows that the neurotoxic effect of H_2_O_2_/Cu(II) on spontaneous spike electrogenesis of the Retzius neurons was reduced in the presence of dimethylthiourea (DMU; 1 mM) and dimethyl sulfoxide (1%), but not mannitol (5 mM).

**Fig. 6.**
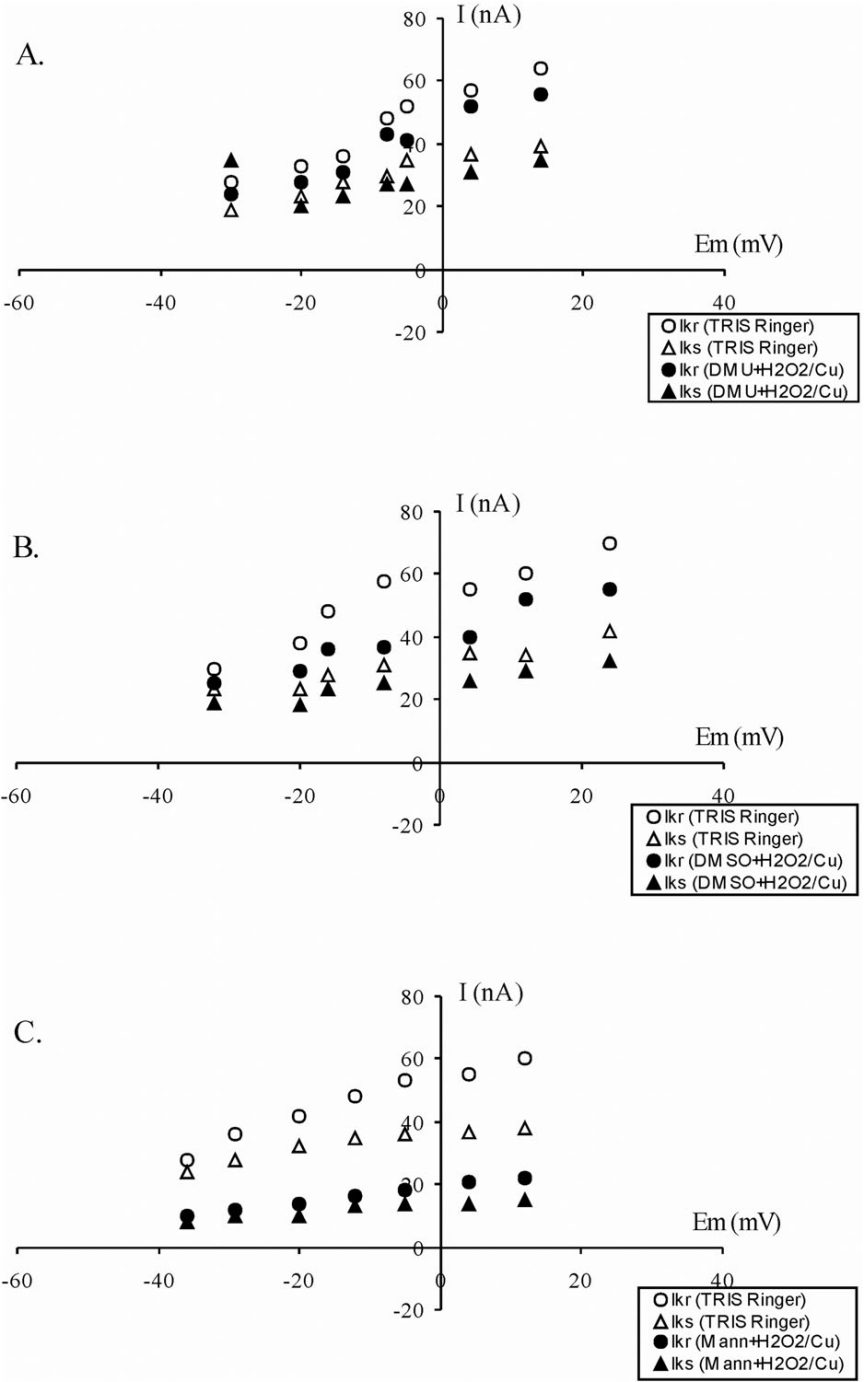
**The effect of the antioxidants on H_2_O_2_/Cu(II)-induced inhibition of the outward K^+^ channels.** (A) DMU (1 mM) prevented inhibition of outward K^+^ channels induced by H_2_O_2_/Cu(II). The current-voltage relationship at the peak of the K^+^ outward current (Ikr) and at the end of stimulation (Iks) in the absence (open symbols) and presence (solid symbols) of DMU. (B) Effects of DMSO (1%) on H_2_O_2_/Cu(II)-induced inhibition of the outward K^+^ channels. The current-voltage relationship at the peak of the K^+^ outward current (Ikr) and at the end of stimulation (Iks) in the absence (open symbols) and presence (solid symbols) of DMSO. (C) Effects of mannitol (5 mM) on H_2_O_2_/Cu(II)-induced inhibition of outward K^+^ channels. The current-voltage relationship at the peak of the K^+^ outward current (Ikr) and at the end of stimulation (Iks) in the absence (open symbols) and presence (solid symbols) of mannitol. Ikr-rapid outward K^+^ current; Iks-slow outward K^+^ current.

In the presence of DMU (1 mM), 1 mM H_2_O_2_ and 0.02 mM Cu(II) produced only a weak reduction of fast and slow potassium outward current of 12.5% and 10.26%, respectively (Fig. 6A), which was significantly smaller than the reduction of 70.15% and 69.24% in the absence of DMU. Pretreatment with another scavenger, DMSO (1%), partially blocked the effect of the H_2_O_2_/Cu(II) oxidation system on the outward K^+^ currents. Voltage clamp experiments revealed that 1 mM H_2_O_2_ and 0.02 mM Cu(II) in the presence of DMSO reduced the outward potassium current by 22%, respectively (Fig. 6B). At the test potential of +24 mV, the fast and slow part of the K^+^ outward current dropped by 21.43% (compared to 70.15% in the absence of the DMSO) and 23.81% (compared to 69.24% in the absence of the DMSO). In the final experiments, Retzius neurons were pretreated with mannitol (5 mM). In contrast to DMU and DMSO, mannitol did not prevent reduction of the outward K^+^ current induced by H_2_O_2_ and Cu(II) (Fig. 6C). In the presence of mannitol, 1 mM H_2_O_2_ and 0.02 mM Cu(II) caused strong inhibition of fast and slow components of 63.34% and 60.53%, respectively (compared with the inhibition of 70.15% and 69.24% in the absence of mannitol).

## DISCUSSION

The main finding of the present study is the determination of the early alteration in electrophysiological properties of Retzius neurons, i.e. membrane potential, action potential and outward repolarizing K^+^ currents. Oxidative stress induced by H_2_O_2_/Cu(II) produced a slow and continuous depolarization of the membrane potential. These results concur with those found by Nakaya et al. (1992) in a study with cardiac cells, in which they proposed that depolarization was partly a consequence of the inhibition of activity in the K^+^ channels. In contrast, Ostrowski et al. (2014) observed that H_2_O_2_ induced hyperpolarization of resting membrane potential of nucleus tractus solitarii neurons. Similar findings have been reported in rat CA1 pyramidal neurons where 3.3 mM of H_2_O_2_ caused a membrane hyperpolarization by inducing an increase in potassium conductance (Seutin et al., 1995). Bychkov et al. (1999) demonstrated that H_2_O_2_ induced both depolarization and hyperpolarization of the membrane potential via two various mechanisms. Low concentrations of H_2_O_2_ elicited membrane potential depolarization, mediated through the inhibition of inward-rectifying K^+^ channels, whereas higher H_2_O_2_ increased the amplitude of the Ca^2+^-activated K^+^ current and thus induced hyperpolarization.

We observed that the amplitude of action potentials decreased, while duration increased in a progressive way along the drug exposure time. Similar data were found in studies on rat intrinsic cardiac ganglia (Whyte et al., 2009) and cortical pyramidal neurons (Pardillo-Díaz et al., 2015) from the effects of ROS donors. The study by Du et al. (2000) suggests that down-modulation of delayed rectifier potassium currents in hippocampal neurons results in the prolongation of action potentials. Hasan et al. (2013) identified HO^•^ as the intermediate oxidant responsible for H_2_O_2_-induced inhibition of the delayed rectifier K^+^ current in the hippocampus through the oxidation of sulfhydryl groups of intracellular cysteine residues. Studies carried out on guinea pig ventricular myocytes have revealed that H_2_O_2_ prolongs action potentials by increasing the late sodium current (Song et al., 2006). In a study on CA1 hippocampus neurons, Angelova and Müller (2006) demonstrated oxidative inhibition of the voltage-gated transient (I_A_) and delayed rectifier [I_K(V)_] potassium currents by H_2_O_2_.

The accumulating evidence suggests that ROS modulate neuronal excitability. It has been shown that combined application of H_2_O_2_ and Cu(II) disrupts both the intrinsic excitability of the neuron and the ability of generating action potentials. These findings are consistent with previous research performed in the CA1 area of the hippocampus, where H_2_O_2_ suppressed neuronal activity, and this effect was emphasized by co-application of H_2_O_2_ and Fe^2+^, proposing that the HO^•^ produced by the Fenton reaction mediates the action of H_2_O_2_ on hippocampal neuronal activity (Garcia et al., 2011). This result goes in line with the one obtained by Pardillo-Díaz et al. (2015) in which cumene hydroperoxide (10 µM; 30 min) produced a complete cancellation of discharge properties in the pyramidal neurons. In contrast, Ostrowski et al. (2014) observed, initially, a decrease of discharge of nucleus tractus solitarii neurons that is followed by sustained hyperexcitability. A study carried out on myenteric neurons (Pouokam et al., 2009) has found that H_2_O_2_ inhibited the Na^+^ channels, hyperpolarized the cell membranes and increased the cytosolic Ca^2+^ concentration. In our study, the HO^•^ seems to be involved in the mechanism of the H_2_O_2_/Cu(II) action. Scavenging of this radical by dimethylthiourea and dimethyl sulfoxide diminished the inhibitory effect of the H_2_O_2_/Cu(II) on the K^+^ channels (Fig. 5). In principle, the hydroxyl radical can induce either direct modulation of the K^+^ channels or indirect via channel-associated regulatory proteins*.*

Voltage-clamp experiments in the present study revealed a strong reduction of the outward K^+^ current. Outward ion currents responsible for repolarization the membrane potential use separate voltage-dependent and Ca^2+^-dependent potassium channels. Electrophysiological studies have shown that there are three classes of K^+^ channels in Retzius nerve cells of the leech: (1) the fast, (2) the slow Ca^2+^-activated K^+^ channel, (3) the late voltage regulated K^+^ channels (Beleslin et al., 1988). According to Stewart et al. (1989) in Retzius cells three principal K^+^ currents have been identified: (1) a transient, inactivating A-type current (I_A_), (2) a Ca^2+^-activated K^+^ current (I_C_), (3) the delayed rectifier currents (I_K1_ and I_K2_). Outward repolarizing K^+^ currents play a fundamental role in determining neuronal excitability and action potential duration. Normally, Retzius nerve cells are spontaneously active, firing at a quite regular rate of 0.2-3 APs/s (Beck et al., 2001). Oxidative modifications of outward K^+^ channel activity lead to changes in action potential duration and the spontaneous electrical activity.

There is accumulating evidence that regulation of ion channels by cellular redox potential may be a significant determinant of channel activity. Nevertheless, the studies have given contradictory results on whether oxidative modification increases or decreases the ion channel activity. Several studies have found that H_2_O_2_ enhanced the Ca^2+^-activated K^+^ channel activity (Bychkov et al., 1999; Dong et al., 2008; Huang et al., 2009b; Feng et al., 2012; Zhang et al., 2012). Other studies reached to the opposite conclusions (DiChiara and Reinhart, 1997; Zhang et al., 2006). DiChiara and Reinhart (1997), for example, reported that the oxidizing agent H_2_O_2_ decreases the activity of the human brain Ca^2+^-activated K^+^ channels (*hslo*), whereas the reducing agent DTT enhances and stabilizes the activity of K^+^ channels. Liu et al. (2009), investigated the effect of H_2_O_2_ on the ‘big conductance’ (BK) channels using the patch-clamp technique. They showed that BK channel activity was inhibited in inside-out patches, whereas it was increased in cell-attached configuration. Soto et al. (2002) found that the H_2_O_2_-induced oxidative modification of the Ca^2+^-dependent K^+^ channels was mediated by the formation HO^•^ and identified cysteine residues (probably located on the cytosolic side of the channel protein) as one of the targets responsible for channel inhibition. These contrary data might be due to the type channels or the type ROS generating system. However, oxidative modification of the K^+^ channels is a complex process that is not fully understood.

The effect of H_2_O_2_/Cu(II) on the electrical properties of Retzius nerve cells in our study, seemed to be mediated by the HO^•^, as Cu(II), which leads to the production of this radical from H_2_O_2_, and strongly potentiates the influence of an ineffective concentration (1 mM) of the H_2_O_2_. Previous results showed that the oxidizing agent H_2_O_2_ did not significantly change the electrical properties of Retzius neurons (Jovanovic and Jovanovic, 2013). To confirm whether the mechanism action of the H_2_O_2_/Cu(II) system on action potential duration and K^+^ channels is in any way attributable to HO^•^, the action of the specific scavengers were investigated. It was indirectly shown that an H_2_O_2_/Cu(II) system elicited oxidative stress, through the examinations with antioxidants where we showed the partial reversal of H_2_O_2_/Cu(II)-induced outward K^+^ channels' inhibition. Also, that the HO^•^ is involved in modification of the electrical properties of Retzius neurons with H_2_O_2_/Cu(II) was demonstrated in the experiments where DMU almost completely prevented and DMSO partially inhibited the effects of H_2_O_2_/Cu(II) on Retzius cells. Mannitol was less effective in prevention of prolongation of action potential duration and inhibition of outward K^+^ channels. Complete protection was not seen with any of the antioxidants used in this study; however, pretreatment with DMU and DMSO significantly reduced the prolongation of action potential duration and the inhibition of the outward K^+^ current, while mannitol did not prevent the inhibition of the outward K^+^ current induced by H_2_O_2_/Cu(II).

There are at least two possible explanations for the incomplete recovery of the action potential duration and the outward K^+^ current in the presence of HO^•^ scavengers. One possibility is that H_2_O_2_ in reaction with Cu(II) ions forms two types of oxidizing species, namely HO^•^, and also some kind of copper-oxygen complex that antioxidants do not scavenge. This agrees with the findings of Kawanishi et al. (2002), who found that the oxidative DNA damage by the H_2_O_2_/Cu(II) was induced by the generation of ROS, such as HO^•^ and a copper-oxygen complex with similar reactivity to HO^•^. It is possible that the HO^•^ as well as the equally reactive alkoxy radicals produced by the Cu(II)-catalyzed Fenton reaction may be partly responsible for the observed prolongation of action potential duration and for the inhibition of the outward K^+^ channels in our study. The other explanation for the incomplete protection of the action potentials and outward K^+^ channel in the presence of HO^•^ scavengers is that a large portion of the oxidants formed are site-specific, i.e. the metal ion producing the radical is attached to ion channel proteins, in such a way that a scavenger has no possible chance of interfering before the radical has hit the target. Several previous studies have reported that DMSO exhibits dual behavior: as an antioxidant and proxidant. DMSO is an antioxidant able to decrease both protein oxidation and lipid peroxidation in rat brain. It also has the ability to trap hydroxyl and hydroperoxyl radicals (Sanmartín-Suárez et al., 2011). However, DMSO also revealed pro-oxidant characteristics, which is a result of the specific activity of DMSO with thiol groups in proteins (Liu et al., 2009). In this study mannitol did not show protective effects, which is probably related to its low permeability and its limited distribution to sites where HO^•^ is generated. The most likely explanation is that some ROS produced from H_2_O_2_/Cu(II) are not neutralized by this antioxidant. The study by Winter et al. (2005) suggests that mannitol is the relatively weak HO^•^ scavenger, which does not have adequate access to the reactive oxygen metabolites.

According to the obtained results, we concluded that the prolongation of the action potentials of Retzius neurons is the result of the effect of the H_2_O_2_/Cu(II) system on outward K^+^ channels. Outward repolarizing K^+^ currents are critical determinants of membrane excitability and action potential firing in neuronal cell (Duprat et al., 1995). Accordingly, oxidative modifications of K^+^ channels results in brain hyperexcitability and eventually cell death. Studies have shown that ROS-mediated oxidation of K^+^ channels is a cause of reduced cognitive function during normal aging (Alshuaib et al., 2001; Cotella et al., 2012; Wu et al., 2013) and neurodegenerative diseases including Alzheimer's and Parkinson's (Cai and Sesti, 2009).

The oxidative mechanism proposed in the present study might have a wider importance not only to simple invertebrate systems such as leeches, but also, similarly, to the brains of mammals. A better understanding of oxidative modifications of ion channels may allow the development of new and specific ion channel therapies in the treatment of brain disorders such as neurodegeneration, epilepsy, and pain.

## MATERIALS AND METHODS

### Experimental animals

The experiments were performed on Retzius nerve cells in isolated leech segmental ganglia at room temperature (20-25°C). Leeches of the species *Haemopis sanguisuga* were obtained from local commercial suppliers. All experimental protocols were approved by the Animal Research Ethics Committee (School of Medicine, University of Belgrade, Serbia). The experimental procedure complies with institutional research council guidelines. The leeches were first anaesthetized in 10% ethanol. Then, the ventral nerve cord was removed from the animal in short segments of four ganglia via a ventral longitudinal incision. Dissected segments were immediately transferred to a 2.5 ml plastic chamber containing a leech Ringer solution (for the composition, see Solutions) and fixed by means of fine steel clips. The plastic chamber was then placed in a grounded Faraday cage mounted on a fixed table in a manner that prevents vibrations. Each segmental ganglion contains approximately 200 pairs of neurons. The largest neurons in the leech central nervous system are Retzius cells (40-60 µm in diameter) which exhibit stable resting membrane potential and which are nonbursting neurons with a low spontaneous firing rate. It is well known that the resting potential of Retzius nerve cells of medical and horse leeches is lower then in other neurons. The resting potential of the Retzius cells ranges from −40 to −60 mV (Lent, 1977; Beleslin et al., 1988; Angstadt, 1999) and the action potentials were generally between 20 and 50 mV and did not overshoot. Because Retzius cells are large and easily identifiable they must be among the most thoroughly investigated single nerve cells (Lent, 1977).

### Electrophysiological methods

Transmembrane action potentials were recorded with conventional microelectrode techniques. Isolated cells were impaled with glass microelectrodes pulled from 1.5 mm borosilicate glass (1.5 mm outside diameter, 0.6 mm inside diameter, Clark Electromedical Instruments, Edenbridge, UK) and filled with a 3 M KCl to give final resistances of 15-20 MΩ. A microelectrode was dipped into the solution and 20-30 min were allowed for equilibration. The recordings were amplified using a Bioelectric Instrument DS2C high input resistant amplifier. Microelectrodes were connected to the amplifier via an Ag-AgCl junction. The ground electrode was an Ag-AgCl wire in a separate chamber filled with Ringer solution connected to the experimental chamber by a 3 M KCl 3% agar bridge.

Activity K^+^ channels were studied in the Retzius nerve cells by using the voltage-clamp technique. Long-lasting depolarizing pulses (to 300 ms) in sodium free Tris Ringer in the neurons where the holding potential was more negative than −40 mV induced a progressive decay of the outward current. The data were leak corrected by using hyperpolarizing pulses of equal magnitude and by assuming a constant leak conductance. Command pulses were derived from a Tektronix 161 pulse generator. Voltage and current records were displaced on a Tektronix 564 oscilloscope. Data were acquired by a Digidata 1200 analog-to-digital board (Axon Instruments, Jakarta, Indonesia), and stored for analysis in a computer. Duration of an action potential was determined at the 90% level of repolarization. Amplitude was the voltage increment between the resting level and spike voltage peak.

### Solutions

Leech Ringer solution composed of (mM): NaCl, 115; KCl, 4; CaCl_2_, 2; Na_2_HPO_4_, 1.2; NaH_2_PO_4_, 0.3 (pH 7.2). In the Na^+^-free Ringer, 115 mM NaCl was completely replaced with an equal amount of Tris (hydroxymethyl) aminomethane-Cl (Tris Ringer), and Na_2_HPO_4_ and NaH_2_PO_4_ were omitted. Pharmacological agents were prepared and dissolved immediately before application in the physiological salt solution at the concentrations stated. H_2_O_2_-containing solutions were prepared fresh, just before each experiment by dilution of a 30% H_2_O_2_ stock solution (Zorka Pharma, Sabac, Serbia) and added to the Ringer solution (or Tris-Ringer solution) at a final concentration of 1 mM. The CuCl_2_ (Sigma, St. Louis, MO, USA) concentration was 0.02 mM. The mannitol, dimethylthiourea and dimethyl sulfoxide were obtained from Sigma, and added to the Ringer solution at final concentrations of 5 mM (mannitol), 1 mM (dimethylthiourea) and 1% (dimethyl sulfoxide). The Retzius nerve cells were treated for 20 min with H_2_O_2_/Cu(II) in the presence or absence of mannitol, dimethylthiourea and dimethyl sulfoxide. To change solutions the chamber was flushed continuously with a volume of fluid at least 10 times that of the chamber volume.

### Statistical analysis

Results are given as mean±standard deviation (s.d.) with the number (*n*) of investigated neurons. Statistical analysis was made using Student's *t*-test. *P* values <0.05 were considered significant.
